# Phenothiazinium Photosensitizers Associated with Silver Nanoparticles in Enhancement of Antimicrobial Photodynamic Therapy

**DOI:** 10.3390/antibiotics10050569

**Published:** 2021-05-12

**Authors:** Glaucia Rigotto Caruso, Ludmilla Tonani, Priscyla Daniely Marcato, Marcia Regina von Zeska Kress

**Affiliations:** 1 Department of Clinical Analysis, Toxicology and Food Sciences, School of Pharmaceutical Sciences of Ribeirao Preto, University of Sao Paulo, Ribeirao Preto 15040-903, SP, Brazil; g_rigotto@hotmail.com (G.R.C.); ludmilla@fcfrp.usp.br (L.T.); 2 Department of Pharmaceutical Sciences, School of Pharmaceutical Sciences of Ribeirao Preto, University of Sao Paulo, Ribeirao Preto 15040-903, SP, Brazil; pmarcato@fcfrp.usp.br

**Keywords:** antimicrobial photodynamic therapy, phenothiazinium, silver nanoparticles, *Candida albicans*, *Fusarium keratoplasticum*

## Abstract

Antimicrobial photodynamic therapy (APDT) and silver nanoparticles (AgNPs) are known as promising alternatives for the control of microorganisms. This study aims to evaluate the antifungal activity of APDT, particularly by using the association of low concentrations of phenothiazinium photosensitizers (PS) methylene blue (MB), new methylene blue N (NMBN), and new methylene blue N Zinc (NMBN-Zn) in association with biosynthesized AgNPs. The AgNPs were characterized by UV-Vis spectrophotometry, transmission electron microscopy, and the dynamic light scattering method. The minimum inhibitory concentration of compounds in APDT against *Candida albicans* and *Fusarium keratoplasticum* was obtained and the Fractional Inhibitory Concentration Index determined the antifungal effect. The toxicity of compounds and associations in APDT were evaluated in *Galleria mellonella*. The AgNPs presented a surface plasmon band peak at 420 nm, hydrodynamic diameter of 86.72 nm, and zeta potential of −28.6 mV. AgNPs-PS showed a wider and displaced plasmon band peak due to PS ligands on the surface and decreased zeta potential. AgNPs-NMBN and AgNPs-NMBN-Zn associations presented synergistic effect in APDT with 15 J cm^−2^ against both fungi and did not show toxicity to *G. mellonella*. Hence, the enhancement of antifungal activity with low concentrations of compounds and absence of toxicity makes APDT with AgNPs-PS a promising therapeutic alternative for fungal infections.

## 1. Introduction

Fungal diseases are globally and gradually on the rise. The indiscriminate and prolonged use of antifungal agents, inappropriate prescription, and interruption of antimicrobial treatment results in the growing increase in drug-resistant microorganisms, including the development of multidrug-resistant strains capable of aggravating patients’ conditions [1,2,3]. The emergence of resistance to commercial antifungals generated the need to study therapeutic alternatives that are low-cost, efficient, and non-toxic to the environment and humans [4]. Thus, antimicrobial photodynamic therapy (APDT) is considered an interesting alternative for the control of pathogenic fungal agents [5,6]. The efficiency of APDT is influenced by factors such as light, type of photosensitizer (PS), selectivity, and production of reactive oxygen species (ROS). Moreover, an amount of PS molecules in an excited triplet state is required for the production of ROS and the consequent inactivation of microorganisms [7,8]. One of the great challenges in APDT is to correctly address the photosensitizers (PS) to increase the effectiveness of APDT using the lowest concentration of PS, the shortest time of light exposure, and light in the therapeutic window [8]. Silver nanoparticles (AgNPs) have shown to be efficient when complexed with photosensitizers, by addressing them correctly and improving the efficiency of APDT [9]. They play an important role in the biomedical field as vehicles for drug delivery, bioimaging, and due to their antimicrobial effect. Their potential is used against multi-resistant bacteria and fungi that may cause serious infection in humans, animals, and plants. AgNPs present unique physical, chemical, and biological characteristics such as resistance, optical properties, high reactivity, and specific interactions with biomolecules and microorganisms [10,11]. The biological method uses living systems capable of producing AgNPs in a clean, non-toxic, and sustainable way. Bacteria, algae, yeasts, and fungi have a great potential to reduce metal ions, so they can agglomerate atoms and synthesize eco-friendly nanoparticles [12].

The limitation of available antifungal drugs makes it crucial to find alternative therapies for fungal infections. Therefore, the objective of the present study is to evaluate the antifungal activity effect of APDT against the yeast fungus *C. albicans* and the filamentous fungus *F. keratoplasticum* particularly by using low concentrations of phenothiazinium photosensitizers (PS) methylene blue (MB), new methylene blue N (NMBN), and new methylene blue N Zinc (NMBN-Zn) in association with biosynthesized AgNPs. The association of PS and AgNPs in APDT was chosen hypothesizing that a synergistic effect against such pathogens might be produced.

## 2. Results

### 2.1. Biosynthesis and Characterization of Silver Nanoparticles

**Figure 1:**
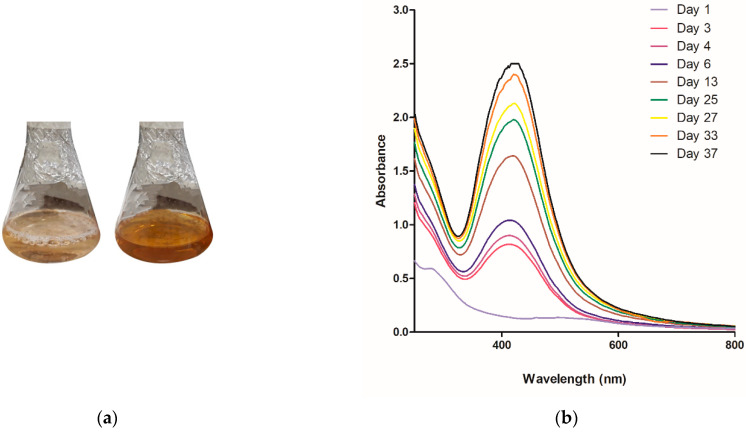

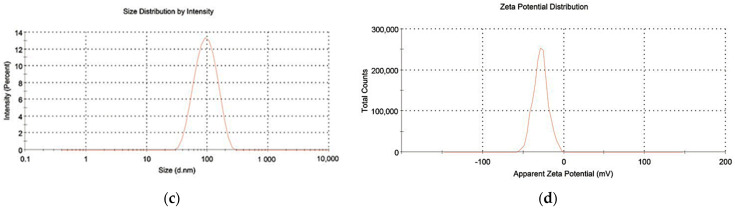
AgNPs biosynthesized by *Fusarium oxysporum*. (**a**) Fungal filtrate on the 1st day (left) and 15th day (right) after addition of silver nitrate (AgNO_3_); (**b**) UV-Vis absorption spectrum of fungal filtrate after addition of AgNO_3_; (**c**) AgNPs size distribution by intensity (Z-average 86.72 nm); and (**d**) Zeta potential distribution (−28.6 mV).

The extracellular biosynthesis of AgNPs was carried out and the formation of AgNPs was monitored by a visual color change of the reaction mixture, as a result of the surface plasmon resonance phenomenon. The color of the reaction mixture began to change on the third day and had changed from slight yellow to brown within 15 days of reaction (Figure 1a). The gradual biosynthesis of AgNPs was confirmed by UV-Vis spectral scanning at 200–800 nm. An increase in plasmon band absorbance was observed at around 420 nm (Figure 1b).

The hydrodynamic diameter of AgNPs was 86.72 nm and the polydispersity index (PdI) was low (less than 0.3), indicating a formulation with low polydisperse nanoparticles (Figure 1c). In addition, AgNPs showed a negative zeta potential (−28.6 mV) (Figure 1d).

**Figure 2:**
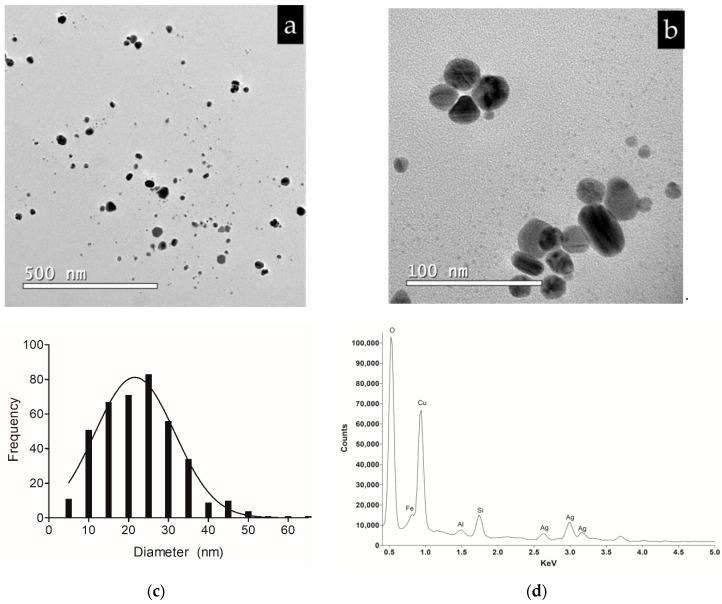
Micrographs of biosynthesized AgNPs obtained by TEM. (**a**) Distribution of AgNPs on a scale of 500 nm; (**b**) AgNPs on a scale of 100 nm with morphology mostly spherical. (**c**) Histogram of the size distribution of AgNPs according to micrographs obtained by TEM (n = 403). (**d**) Energy dispersive X-ray (EDX) spectrum of AgNPs.

The transmission electron microscopy (TEM) micrographs presented a heterogeneous size distribution, mostly spherical and measuring 16–33 nm (Figure 2a–c). The presence of an elemental silver signal in the colloidal dispersion was confirmed by Energy dispersive X-ray (EDX) spectrum (Figure 2d).

### 2.2. The Association of AgNPs with MB, NMBN, and NMBN-Zn

**Figure 3:**
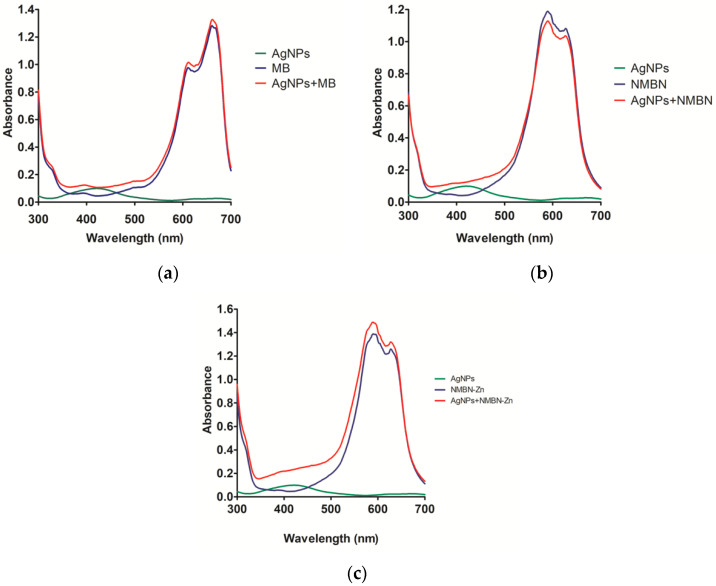
Optical absorption spectrum of AgNPs-PS associations compared to the isolated compounds. (**a**) AgNPs (1.51 µg mL^−1^), MB (6.39 µg mL^−1^), and AgNPs-MB (1.51 µg mL^−1^ and 6.39 µg mL^−1^); (**b**) AgNPs (1.32 µg mL^−1^), NMBN (6.95 µg mL^−1^), and AgNPs-NMBN (1.32 µg mL^−1^ and 6.95 µg mL^−1^); and (**c**) AgNPs (2.56 µg mL^−1^), NMBN-Zn (8.33 µg mL^−1^), and AgNPs-NMBN-Zn (2.56 µg mL^−1^ and 8.33 µg mL^−1^).

The AgNPs-PS were characterized by variations in the formation of the surface plasmon band. The absorption graphs show curves of AgNPs, PS (MB, NMBN, and NMBN-Zn), and also of AgNPs-PS (Figure 3a–c).

The compounds (green and blue curves) and AgNPs-PS association (red curves) showed the presence of AgNPs through the altered plasmon band around 420 nm and the respective PS in their maximum absorption peaks (MB, 655 nm; NMBN 624 nm; NMBN-Zn 627 nm) (Figure 3a–c). The optical absorption spectrum graphs of AgNPs-PS presented an extension in the plasmon band, indicating that the presence of PS altered the electron distribution on the AgNPs surface. Zeta potential was measured to assess the influence of the presence of PS associated with AgNPs. As a result, it was observed that the zeta potential of AgNPs-MB, AgNPs-NMBN, and AgNPs-NMBN-Zn were −13.9 mV, −17 mV, and −9.25 mV, respectively, meaning that zeta potential decreased due to the presence of cationic phenothiazinium photosensitizers.

### 2.3. Antifungal Activity of AgNPs-PS in APDT

The antifungal activity of APDT with each PS (MB, NMBN, and NMBN-Zn), biosynthesized AgNPs, and AgNPs-PS (AgNPs-MB, AgNPs-NMBN, and AgNPs-NMBN-Zn) was evaluated in the dark and combined with different radiant exposures of red light (5, 10, and 15 J cm^−2^). Exposure to red light (at all radiant exposures) in the absence of the PS and AgNPs did not inhibit the growth of *C. albicans* and *F. keratoplasticum* (data not shown). The minimum inhibitory concentration (MIC) value of AgNPs at 0, 5, 10, and 15 J cm^−2^ were 1.56 µg mL^−1^ and 6.25 µg mL^−1^ against *C. albica*ns and *F. keratoplasticum*, respectively. The AgNPs present an absorption peak around 420 nm and the antifungal effect is not influenced by red light (emission spectrum between 600 and 650 nm), justifying the same MIC for dark and all radiant exposures. The treatment in the dark with the highest concentration of MB (>12.79 µg mL^−1^), NMBN (>13.91 µg mL^−1^), and NMBN-Zn (>16.66 µg mL^−1^) did not inhibit the growth of either fungus. The radiant exposure of 15 J cm^−2^ showed the best conditions for APDT with MB, NMBN, and NMBN-Zn antifungal activity. Additionally, it was noted that the greater the radiant exposure, the lesser the amount of PS needed to inhibit the growth of microorganisms (Table 1 and Table 2).

The association of PS with AgNPs was carried out with a concentration gradient based on the MIC of isolated PS and AgNPs against *C. albicans* and *F. keratoplasticum*. They were mixed in different concentrations particularly aiming to enhance the antifungal activity of APDT by using low amounts of the compounds (Table 1 and Table 2). For *C. albicans*, a synergistic effect was observed in AgNPs-NMBN with a fractional inhibitory concentration index (FICI) of 0.26 (10 J cm^−2^) and 0.5 (15 J cm^−2^); and AgNPs-NMBN-Zn presented FICI of 0.5 (10 J cm^−2^) and 0.35 (15 J cm^−2^) (Table 3). For *F. keratoplasticum*, AgNPs-NMBN also presented a synergistic effect with FICI of 0.3 (10 J cm^−2^) and 0.5 (15 J cm^−2^); and AgNPs-NMBN-Zn presented FICI of 0.18 (5 J cm^−2^) and 0.31 (10 J cm^−2^) (Table 4) [13,14].

**Figure 4:**
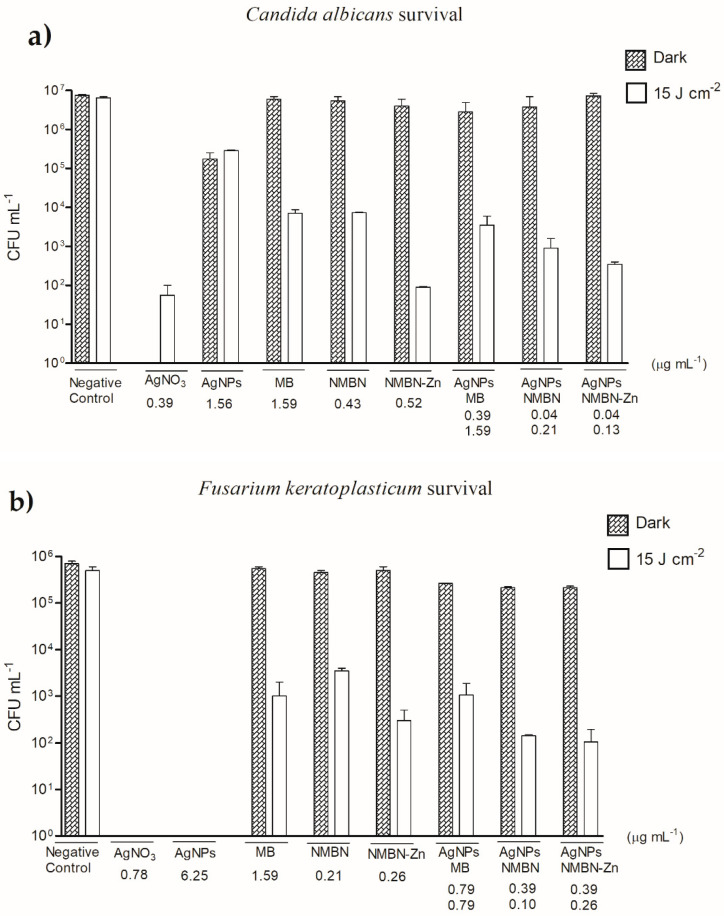
Antifungal survival test. The effect of APDT with the radiant exposure of 15 J cm^−2^ on the survival of the yeasts of *C. albicans* (**a**) and filamentous fungus *F. keratoplasticum.* (**b**) The concentrations of the compounds are shown in Table 1 and Table 2. Error bars are the standard deviation of three independent experiments. CFU, colony-forming units; Negative control, dark and red light (15 J cm^−2^); AgNO_3_, silver nitrate; AgNPs, silver nanoparticles; MB, methylene blue; NMBN, new methylene blue N; NMBN-Zn, new methylene blue N Zinc.

Survival of *C. albicans* and *F. keratoplasticum* were tested in APDT in the dark (control) and red light (15 J cm^−2^). The concentrations of each compound were based on the respective MIC found in the susceptibility test with a radiant exposure of 15 J cm^−2^ (Table 1 and Table 2). The red light did not affect *C. albicans* survival, and the number of surviving yeasts was close to the initial inoculum (1 × 10^7^ CFU mL^−1^). As expected, AgNO_3_ in both dark and red light killed most of the yeasts and only 1 × 10^2^ CFU mL^−1^ survived (Figure 4a). AgNPs showed fungal survival of 1 × 10^5^ CFU mL^−1^ in both dark and red light. As result, it was observed that in the dark, both isolated PS and those associated with AgNPs did not kill the yeasts. In the presence of red light, both PS and the associations showed a significant reduction in survival of *C. albicans* (*p* < 0.05) (Figure 4a). Therefore, the concentrations of PS and AgNPs-PS that inhibited the growth (MIC) of the fungi in APDT were also able to kill *C. albicans* yeasts.

The red light did not affect *F. keratoplasticum*, and the number of surviving microconidia was close to the initial inoculum (1 × 10^6^ CFU mL^−1^). In the presence of AgNO_3_ and AgNPs in both dark and red light (15 J cm^−2^), there were no survivors (Figure 4b). As a result, it was observed that in the dark, both PS isolated and PS associated with AgNPs did not kill microconidia. In the presence of red light, both PS and AgNPs-PS showed a significant reduction in the survival of *F. keratoplasticum* microconidia (*p* < 0.05) (Figure 4b). Therefore, the concentrations of PS and associations that inhibited the growth (MIC) of the fungi in APDT were also able to kill *F. keratoplasticum* microconidia.

**Figure 5:**
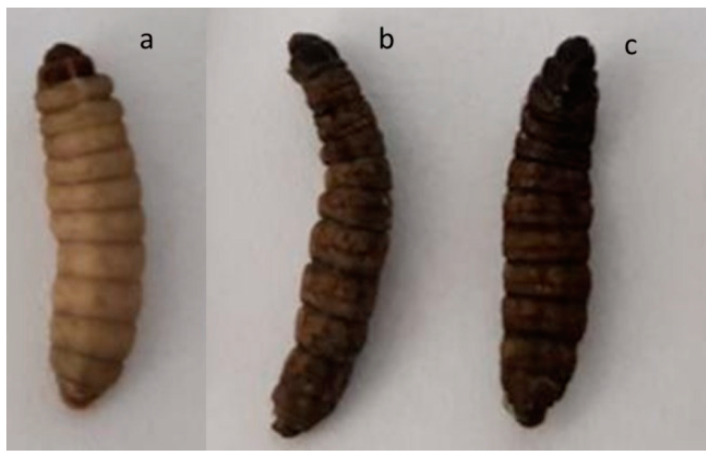
*Galleria mellonella* larvae. (**a**) Healthy larva without treatment (naïve); (**b**) dead larvae after AgNO_3_ in exposure to dark; (**c**) dead larvae after AgNO_3_ in exposure to red light (15 J cm^−2^).

**Figure 6:**
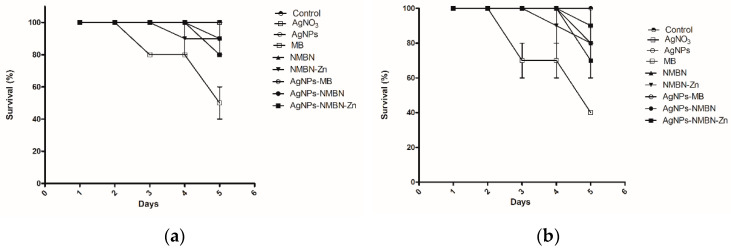
Toxicity of APDT in *G. mellonella* larvae. Larvae were inoculated with the compounds AgNO_3_, AgNPs, MB, NMBN, NMBN-Zn, and associations. The concentration of each compound injected in *G. mellonella* larvae is shown in Table 5, group of the larvae number 5. (**a**) Survival of *G. mellonella* larvae over the days in the dark; (**b**) survival of *G. mellonella* larvae over the days after APDT 15 J cm^−2^. Control, naïve and PBS; AgNO_3_, silver nitrate; AgNPs, silver nanoparticles; MB, methylene blue; NMBN, new methylene blue N; NMBN-Zn, new methylene blue N Zinc; associations AgNPs-MB, AgNPs-NMBN, and AgNPs-NMBN-Zn.

The toxicity of APDT with AgNO_3_, AgNPs, PS (MB, NMBN, and NMBN-Zn), and the AgNPs–PS (AgNPs-MB, AgNPs-NMBN, and AgNPs-NMBN-Zn) was evaluated with larvae of *Galleria mellonella*, an invertebrate and non-rodent model of virulence and toxicity. Table 5 shows the doses of each compound injected in *G. mellonella*. All control larvae of the experiment (naïve and phosphate buffered saline-PBS) exposed to dark and red light (15 J cm^−2^) survived until the end of the experiment (Figure 5a and Figure 6). The positive control (AgNO_3_) showed a gradual increase in death of larvae with the increase in the compound concentration, in exposure to both dark and red light (Figure 5b,c, Supplementary Figure S1). The highest concentration of AgNO_3_ reduced the larvae population to 40% in 5 days (Figure 6). The dark and red light (15 J cm^−2^) treatments with AgNPs, MB, NMBN, NMBN-Zn, and associations (AgNPs-MB, AgNPs-NMBN, and AgNPs-NMBN-Zn) did not present toxicity to *G. mellonella* larvae at the maximum tested doses for AgNPs (5 mg kg^−1^), MB (20.83 mg kg^−1^), NMBN (26.31 mg kg^−1^), and NMBN-Zn (16 mg kg^−1^) (Figure 6, supplementary Figure S1).

## 3. Discussion

The frequency of mycoses due to opportunistic and multi-resistant fungal pathogens has increased significantly over the past decades [1,3]. The field of medical mycology therapy has become an extremely challenging study, making the search for new alternatives for the treatment of fungal infections important. APDT and AgNPs are promising alternative therapies due to their well-known antimicrobial activity. In this context, we studied the antifungal activity effect of APDT against the yeast fungus *C. albicans* and the filamentous fungus *F. kertoplasticum* to find synergistic effect using low concentrations of phenothiazinium photosensitizers (PS) methylene blue (MB), new methylene blue N (NMBN), and new methylene blue N Zinc (NMBN-Zn) in association with biosynthesized AgNPs.

Each PS (MB, NMBN, and NMBN-Zn) has high selectivity for *F. keratoplasticum* and *C. albicans* [6,15]. Additionally, the relationship of metal ions such as Zn^2+^ and Al^3+^ positively influences the photodynamic effect, increasing the production of PS molecules in triplet state and production of singlet oxygen [16,17]. Recently, metallic nanoparticles associated with photosensitizers were explored. Gold nanoparticles associated with toluidine blue (TBO) increased the bactericidal effect of APDT against *Staphylococcus aureus* by 90 to 99% [18,19]. AgNPs associated with TBO against *Streptococcus mutans* also increased bactericidal effect [9,20]. The use of *F. oxysporum* for AgNPs biosynthesis is well described [21,22,23,24]. The biomolecules secreted by the microbial biomass can act as reducing and capping agents during the synthesis of metallic nanoparticles [25]. The relation between biomass composition and metal nanoparticle formation is not clearly elucidated and described in the literature. However, it has been previously verified that different fungi produce reducing agents (e.g., naphthoquinone anthraquinones) and reductase enzymes capable of transferring electrons to silver ions (Ag^+^) [22,24]. Although biomass analysis was not performed in this work, the presence of several intra and extracellular proteins and compounds from *F. oxysporum* (e.g., beta-glucosidase, beta-amylase, peroxiredoxin [26], glycoproteins (60–70 kDa) [27], cationic proteins (24 and 28 KDa) [28], reductases [26], calnexin [29], anthraquinones [30]) has been described. The presence of proteins, hydrolytic enzymes, and reductases depends on which source of nitrogen is used. Therefore, nutrients are important factors that must be studied to improve the quality and efficiency of AgNPs production in the future [31].

The first characteristic observed in AgNPs biosynthesis was the change in dispersion color from yellow to brown and the presence of a band at 420 nm that occurs due to surface plasmon resonance, which is the differentiated movement of electrons in the metal’s electronic bands that are on the surface of nanoparticles [32,33]. The wavelength of the plasmon band is closely related to the size, shape, and distribution of compounds bound to the surface of the AgNPs. Thus, the wider the peak of the plasmon band, the higher the PdI; and the greater the wavelength, the larger the size of AgNPs. Mie’s theory [34] suggests spherical-shaped nanoparticles have a single peak in the absorption spectrum, while particles of other shapes such as triangular or flat disc-shaped may have two or more bands due to their shape and diameter. Based on this theory, the biosynthesis of nanoparticles by *F. oxysporum* showed a single narrow peak located at the 420 nm wavelength, which means spherical AgNPs with an approximate size of 30 nm. Additionally, these data corroborate with results of dynamic light scattering (DLS) and the PdI. Through TEM analysis, it was possible to obtain the average diameter of AgNPs, of which 80% are between 16 and 33 nm. The difference in the size of AgNPs between the DLS and TEM technique is justified by the DLS technique to measure the size of AgNPs in aqueous dispersion, which may be larger due to other compounds from biological synthesis (proteins, for example) that can be bound to nanoparticles [35,36]. Marcato et al. (2015, 2012) and Birla et al. (2013) verified, by Fourier Transform Infrared Spectroscopy (FTIR), the presence of protein capping around AgNPs biosynthesized by *F. oxysporum* [37,38,39]. AgNPs showed a negative zeta potential (−28.6 mV) due to the fungus protein capping around silver nanoparticles, as also described previously [38,39]. Furthermore, through X-ray Powder Diffraction (XRD), Gaikwad et al. (2013) confirmed that biogenic AgNPs prepared using *F. oxysporum* are crystalline [40].

The wide plasmon band observed in Figure 1b indicates the heterogeneity of the particles that is characteristic of the biological synthesis method [21,22]. This heterogeneity was confirmed by the PdI values in the DLS analysis. In comparison with biosynthesis, chemical synthesis of AgNPs generally results in samples with low PdI values due to the possibility of having greater control over reaction variables, such as reducing agent, salt silver, temperature, pH, and stabilizing agents [41]. However, biosynthesis is more advantageous as it is a simpler and cheaper method that does not use toxic agents and does not require sample purification steps to remove the excess of reagents or residual compounds [42,43]. AgNPs biosynthesized by *F. oxysporum* presented a moderate PdI, possibly due to the presence of different reducing agents excreted in the medium by the microorganism that make synthesis differentiated. Furthermore, microorganisms excrete enzymes that catalyze the reaction and proteins that will stabilize nanoparticles without the need to add stabilizing agents, such as polymers [44,45].

The biosynthesized AgNPs associated to MB, NMBN, and NMBN-Zn showed an absorption spectrum peak around 420 nm, which represents the presence of AgNPs, and an additional peak between 600–650 nm, which shows the presence of PS [18,19]. For AgNPs-PS, a decrease in the intensity and an enlargement of the plasmon band at 420 nm were observed. This is justified by the binding of PS to the surface of AgNPs that changes the movement of electrons and increases the diameter of the nanoparticle. Additionally, a change in zeta potential value was observed in AgNPs-PS, as expected [46].

The antifungal activity of AgNPs disrupts the cell wall and plasma membrane and increases the production of ROS that leads to cell death [47,48,49]. AgNPs showed antifungal activity against *C. albicans* and *F. keratoplasticum*, and the MICs were 1.56 µg mL^−1^ and 6.25 µg mL^−1^, respectively. The MICs of AgNPs in the presence of red light were identical to those found in the absence of light (darkness). This is justified by the fact that the spectrum of red light used in the experiment is in the 600 nm range, with the maximum emission peak at 635 nm [6] while the spectrum absorption rates of AgNPs are around 420 nm. The photosensitizers MB and NMBN-Zn have antifungal activity in vitro and in vivo against *Fusarium* spp. [6,50], and in vitro activity against *Candida* spp. [15]. In this work, antifungal activity was evaluated by determining the MIC to evaluate the enhancement of the antifungal activity of AgNPs-PS associations in APDT compared to the isolated compounds. APDT with PS were tested and the most effective PS in APDT (15 J cm^−2^) against *C. albicans* and *F. keratoplasticum* was NMBN-Zn, followed by NMBN and MB. NMBN-Zn had the smallest MIC and its antifungal effect was described for *Candida* spp., *Trichophyton* spp., *Fusarium* spp., and *Colletotrichum* spp. [15,50,51,52].

The AgNPs-PS were tested for APDT antifungal activity and it was observed that among the three associations, AgNPs-NMBN-Zn was the most efficient against *C. albicans* with a synergistic effect presenting FICI of 0.3. For *F. keratoplasticum*, the most efficient association was AgNP-NMBN with a synergistic effect presenting FICI of 0.5. The efficiency of antifungal activity of APDT associations was also assessed by the survivor fraction method, in which AgNPs-MB, AgNPs-NMBN, and AgNPs-NMBN-Zn presented greater inhibition of *C. albicans* and *F. keratoplasticum*, reducing by approximately four orders of magnitude. The association of phenothiazinium PS with nanoparticles has been studied to increase the photodynamic effect, using them as a vehicle, or to increase the number of PS molecules in the triplet state, transferring energy to them. The use of phthalocyanine associated with gold nanoparticles was described and demonstrated an increase in singlet oxygen compared to free phthalocyanine [53]. A hybrid system of AgNPs, amphiphilic polymer, and hematoporphyrin verified a high efficiency in photodynamic inactivation of *Staphylococcus epidermidis* and *Escherichia coli*, as well as presenting low toxicity in cell cultures [54]. In a study, gold nanoparticles complexed to MB demonstrated that metallic nanoparticles act as a “quencher” for MB fluorescence, and photodynamic reaction also occurs with greater intensity through the type I photoprocess, producing hydroxyl radicals [55]. Thus, the association of PS with AgNPs in APDT is promising for the study and use in photodynamic therapy, aiming to increase its effect mainly in the treatment of fungal infections.

APDT with AgNPs-PS associations were tested for toxicity in vivo in the invertebrate model *Galleria mellonella*. The results obtained in experiments with the larvae reproduce several results obtained with mammals due to the similarity of their cells with the innate immune cells of mammals. The use of this model serves as a screening method for tests to decrease the use of mice and rats [56,57]. Its use has been described to assess virulence in microorganisms such as *Cryptococcus neoformans* [58], *Candida albicans* [59], and *Fusarium* spp. [50]. The biosynthesized AgNPs, the PS (MB, NMBN, and NMBN-Zn), and associations (AgNPs-MB, AgNPs-NMBN, and AgNPs-NMBN-Zn) were not able to kill the larvae at the maximum tested dose in APDT. Thus, APDT using PS associated with AgNPs has great potential for the treatment and control of opportunistic mycoses due to its enhanced antifungal activity against *C. albicans* and *F. keratoplasticum* and low toxicity in an invertebrate model of toxicity.

## 4. Materials and Methods

### 4.1. Materials and Fungal Strains

*Fusarium oxysporum* INCQS 40144 (ATCC^®^ 48112™) obtained from the American Type Culture Collection (ATCC) (Manassas, VA, USA) was used to perform biosynthesis of AgNPs. The fungus was grown on Sabouraud dextrose agar (SDA, Neogen, MI, USA) at 28 °C for 5 days. The APDT experiments were performed with *Fusarium keratoplasticum* INCQS 40099 (ATCC 36031) and *Candida albicans* (ATCC 64548). *F. keratoplasticum* was grown in potato dextrose agar (Neogen, Lansing, MI, USA) at 28 °C for 5 days. The microconidia were subsequently harvested and resuspended in PBS. *Candida albicans* was grown on Sabouraud dextrose agar (SDA—Neogen, Lansing, MI, USA) at 35 °C for 24 h. Subsequently, 3 colonies were inoculated in Sabouraud Dextrose liquid culture medium (Neogen, Lansing, MI, USA) and incubated at 35 °C and 180 rpm (Infors HT Ecotron, Switzerland) for 24 h. The liquid culture medium containing the *C. albicans* was centrifuged and the yeasts were resuspended in PBS. The concentration of microconidia and yeast suspension was determined by hemacytometer counts, and diluted with autoclaved PBS to the desired concentrations.

### 4.2. Biosynthesis and Characterization of AgNPs

*Fusarium oxysporum* biomass was gently harvested from SDA and each 10 g was added to 100 mL of sterile water at 25 °C, 150 rpm for 3 days. The biomass was subsequently removed by filtration and the filtrate was centrifuged at 4,000× *g* to remove the undesirable contaminants. Into 10 mL of supernatant were added 2 mg of silver nitrate (AgNO_3_—Sigma-Aldrich, Inc., St. Louis, MO, USA). The solution was stored in an amber flask at 26 °C ± 2 °C in the dark and the formation of AgNPs was observed by color change.

Absorbance spectra of AgNPs were taken for 37 days with a NanoPhotometer P-Class spectrophotometer (IMPLEN GmbH, Munich, Germany) over the wavelength range 300–800 nm. DLS and Zeta Potential measurements were determined using Malvern Zetasizer Nano-ZS equipment (Malvern, UK). Furthermore, to analyze size and shape, AgNPs were subjected to TEM. The sample was dripped onto a 3 mm diameter copper grid coated with carbon, and TEM micrographs were captured in a JEOL/JEM 2100 LaB6 200 kV instrument (JEOL, Boston, MA, USA). Image J software (ImageJ 1.43a, version 64-bits—National Institutes of Health, Bethesda, MD, USA) was used to measure the AgNPs diameter, and data analyses were performed using GraphPad Prism 5 software.

### 4.3. Antimicrobial Photodynamic Therapy (APDT) with Phenothiazinium Photosensitizers and Association with AgNPs

#### 4.3.1. Photosensitizers

The photosensitizers (PS) used were the commercial phenothiaziniums methylene blue (methylene blue, MB) (C_16_H_18_ClN_3_S), new methylene blue N (new methylene blue N, NMBN) (C_18_H_22_ClN_3_S), and new methylene blue N formula with zinc (new methylene blue N zinc chloride double salt, NMBN-Zn) (C_18_H_22_ClN_3_S·0.5 ZnCl_2_). All dyes were obtained from Sigma-Aldrich, Inc., MO, USA.

#### 4.3.2. Light

The APDT was evaluated with an arrangement of 96 LEDs (red light) with an emission spectrum between 600 and 650 nm (peak at 635 nm). The irradiance measurement was 0.01523 W cm^−2^ and APDT experiments were carried out on radiant exposures of 5, 10, and 15 J cm^−2^, corresponding to the exposure times of 5.47 min, 10.94 min, and 16.41 min, respectively.

#### 4.3.3. The Association of AgNPs with MB, NMBN, and NMBN-Zn

The PS and AgNPs solutions were prepared in autoclaved distilled water at concentrations of 82 µg mL^−1^ for MB, 70 µg mL^−1^ for NMBN, 90 µg mL^−1^ for NMBN-Zn, and 100 µg mL^−1^ for AgNPs. The concentrations for the AgNPs-PS were based on the compound concentrations used in MICs obtained from each compound for *C. albicans* and *F. keratoplasticum*. Thus, the lowest concentrations of PS and AgNPs capable of inhibiting the growth of fungi were mixed to form the AgNPs-PS.

The formation of the surface plasmon band of the AgNPs-PS was determined using the NanoPhotometer P-Class spectrophotometer (IMPLEN GmbH, Munich, Germany). The suspensions of AgNPs-PS were diluted in a proportion of 1:1 in distilled water, added into a quartz cuvette, with an optical path of 1 cm, and scanning analysis from 300 to 800 nm was performed on a NanoPhotometer P-Class (IMPLEN GmbH, Munich, Germany). The behavior of the plasmon band of AgNPs was evaluated after the association with the PS in solution. It was also verified if there were changes in the absorption spectrum of the PS. The equipment reading was blanked with autoclaved distilled water that corresponds to the solvent of the synthesized AgNPs.

The hydrodynamic mean diameter (z-average), zeta potential, and polydispersity index of AgNPs-MB, AgNPs-NMBN, and AgNPs-NMBN-Zn were obtained using the DLS technique on the NanoSize ZS device (Malvern). The solutions were diluted 20× with distilled water in a polystyrene bucket for analysis of the hydrodynamic diameter. To measure the zeta potential, the dilution ratio was the same, but the sample was diluted in 1 mM KCl and added to the zeta potential cuvette (DTS 1070, Malvern).

The synergism between compounds was determined using the Fractional Inhibitory Concentration Index (FICI), in which synergy is less than or equal to 0.5. For values between 0.5 and 1.0, the compounds have additive effects; for values between 1.0 and 4.0, the effect is considered indifferent, and antagonism can be observed when the FICI values are above 4 [13].

#### 4.3.4. Evaluation of APDT Effect on *C. albicans* and *F. keratoplasticum* Based on Minimum Inhibitory Concentration (MIC)

The antifungal activity of APDT with PS (MB, NMBN, and NMBN-Zn), AgNPs, and AgNPs-PS against *C. albicans* and *F. keratoplasticum* was determined by the MIC. The experiment was carried out following the guidelines of the microdilution method in broth protocols M38A2 and M27A3 of the Clinical and Laboratory Standards Institute (CLSI, 2008), with modifications. In a 96-well microplate (TPP, Trasadingen, Switzerland), 50 µL of microconidia or yeast suspension and 50 µL of the PS or AgNPs or AgNPs-PS were pipetted. The final concentration of microconidia was 1 × 10^5^ cells mL^−1^ and 5 × 10^3^ cells mL^−1^ of yeast. The MB concentration gradient was 0.02 to 12.79 µg mL^−1^, NMBN from 0.05 to 13.91 µg mL^−1^, and NMBN-Zn from 0.06 to 13.91 µg mL^−1^ for *F. keratoplasticum* and *C. albicans*. The gradients of AgNPs were 0.01 to 4 µg mL^−1^ for *C. albicans*, and 0.02 to 6.5 µg mL^−1^ for *F. keratoplasticum*. The microplates were kept in the dark for 30 min (pre-incubation) and were then exposed to red light at 5, 10, and 15 J cm^−2^. Subsequently, 100 µL of RPMI 1640 culture medium (Sigma Aldrich, USA) twice concentrated buffered with 0.165 M MOPS (Sigma Aldrich, USA) were pipetted into each well of the 96-well plate. The plates were incubated at 28 °C for *F. keratoplasticum* and 35 °C for *C. albicans* in the dark and the growth of the fungus was evaluated after 24 h of incubation. As a negative control, wells were made with PBS and RPMI 1640 culture medium (Sigma Aldrich, USA), and wells with RPMI 1640 culture medium (Sigma Aldrich, USA) plus PS, AgNPs, and AgNPs-PS. As a positive control, wells were made with RPMI 1640 (Sigma Aldrich, USA) plus cells (yeasts or microconidia). In parallel, a replica plate of the experiment was made without exposure to red light. The experiments were carried out in two biological replicates and in triplicate.

#### 4.3.5. Evaluation of APDT Effect Based on the Survival of *C. albicans* and *F. keratoplasticum*

The survival of *C. albicans* and *F. keratoplasticum* was evaluated after APDT with the following compounds: AgNO_3_, AgNPs, MB, NMBN, NMBN-Zn, AgNPs-MB, AgNPs-NMBN, and AgNPs-NMBN-Zn. To assess cell survival (yeasts or microconidia) to APDT, 300 μL of the compounds were mixed with 300 μL of the cell suspension in 1.5 mL microtubes (polypropylene; Axygen Scientific, CA, USA). For *C. albicans*, the final concentration of yeast suspension was 1 × 10^7^ cells mL^−1^ [15] and the compounds are described in Table 1 and Table 2. For *F. keratoplasticum*, the final microconidium concentration was 2 × 10^6^ cells mL^−1^ [6]. The microtubes were kept in the dark for 30 min at 28 °C (pre-incubation). Subsequently, 600 μL of each mixture were transferred to a 24-well plate well (TPP, Switzerland) and exposed to radiant exposures of 0 (control in the dark) and 15 J cm^−2^. The mixtures were then diluted with PBS, pH 7.0 in concentrations 10^−1^ to 10^−3^. The 50 μL volume of each dilution was spread over the surface of 5 mL of potato dextrose agar, supplemented with 1 g L^−1^ of deoxycholic acid (sodium salt) (Fluka, Milano, Italy), in Petri dishes (60 mm × 15 mm). The plates were incubated in the dark at 37 °C, and colony counting started after 24 h. The controls of the experiment were: cells exposed only to red light without the presence of treatments, and cells with all treatments without exposure to light (dark). Two biological replicates were performed, and for each treatment (mixture) three replication plates were made.

### 4.4. Toxicity test in Galleria mellonella

The toxicity of compounds AgNO_3_, AgNPs, MB, NMBN, NMBN-Zn, and AgNPs-MB, AgNPs-NMBN, and AgNPs-NMBN-Zn were evaluated using larvae from the experimental toxicity model *G. mellonella* [60]. For each treatment, 5 larvae of *G. mellonella* (200 to 250 mg) were separated in the sixth instar of development in Petri dishes (90 mm × 15 mm). Doses are described in Table 5. A Hamilton model 7000.5 KH micro-syringe was used to inject 5 μL of inoculum containing the compounds into the hemocoel of each larva through the last right proleg. After inoculation, the larvae were kept in the dark for 30 min at 28 °C and then exposed to radiant exposures of 0 (dark control) and 15 J cm^−2^. The experiment controls were larvae inoculated with PBS exposed and not exposed to light, and larvae that were not inoculated exposed and not exposed to light (naïve). After APDT, the larvae were incubated at 37 °C and deprived of food and direct lighting. Larvae survival assessments were carried out every 24 h for 5 days and pre-pupae were also removed daily to delay metamorphosis. The experiment was conducted with two biological replicates.

### 4.5. Statistics

ANOVA and frequency distribution were performed using GraphPad Prism 5 statistical software. *p*-value < 0.05 was considered statistically significant.

## 5. Conclusions

The enhancement of the antifungal activity of APDT with AgNPs-PS associations by using low concentrations of phenothiazinium photosensitizers (PS) methylene blue (MB), new methylene blue N (NMBN), and new methylene blue N Zinc (NMBN-Zn) in association with biosynthesized AgNPs against *C. albicans* and *F. keratoplasticum* is shown. The APDT with AgNPs, PS, and AgNPs-PS did not show toxicity at the concentrations tested in the in vivo toxicity model *Galleria mellonella*. Thus, the synergistic effect of AgNPs-PS in APDT and the non-toxicity in an in vivo model indicates this as a promising approach for the treatment of superficial mycoses, such as onychomycoses and dermatomycoses; however, more in vivo studies are needed to ensure safety in their use in humans and animals.

## 6. Patents

BR 10 2020 025170 8 filled in Brazil, on 19 December 2020. Title: “Composições compreendendo fotossensibilizadores fenotiazínicos associados à nanopartículas de prata utilizadas na terapia fotodinâmica antimicrobiana e uso destas na preparação de um medicamento para tratar infecções causadas por fungos”.

## Figures and Tables

**Table 1:** Minimum inhibitory concentration (MIC) of AgNPs (µg mL^−1^), phenothiazinium PS (MB, NMBN, and NMBN-Zn in µg mL^−1^), and AgNPs–PS in antimicrobial photodynamic treatment (APDT) against *C. albicans*.

Compound/ Radiant Exposure	0 J cm^−2^	5 J cm^−2^	10 J cm^−2^	15 J cm^−2^
AgNO_3_	1.56	1.56	1.56	0.39 *
AgNPs	1.56	1.56	1.56	1.56 *
MB	>12.79	12.79	6.39	1.59 *
NMBN	>13.91	1.73	0.86	0.43 *
NMBN-Zn	>16.66	2.08	1.04	0.52 *
AgNPs-MB	1.56/12.79	1.56/12.79	0.78/6.39	0.39 */1.59 *
AgNPs-NMBN	1.56/13.91	1.56/13.91	0.04/0.21	0.04 */0.21 *
AgNPs-NMBN-Zn	1.56/16.66	0.04/0.52	0.04/0.52	0.04 */0.13 *

AgNO_3_, silver nitrate; AgNPs, silver nanoparticles; MB, methylene blue; NMBN, new methylene blue N; NMBN-Zn, new methylene blue N Zinc; *, concentration (µg mL^−1^) used in antifungal survival test.

**Table 2:** Minimum inhibitory concentration (MIC) of AgNPs (µg mL^−1^), phenothiazinium PS (MB, NMBN, and NMBN-Zn in µg mL^−1^), and AgNPs-PS in antimicrobial photodynamic treatment (APDT) against *F. keratoplasticum*.

Compound/ Radiant Exposure	0 J cm^−2^	5 J cm^−2^	10 J cm^−2^	15 J cm^−2^
AgNO_3_	1.56	3.12	3.12	0.78 *
AgNPs	6.25	6.25	6.25	6.25 *
MB	>12.79	3.19	1.59	1.59 *
NMBN	>13.91	0.86	0.86	0.21 *
NMBN-Zn	>16.66	2.08	1.04	0.26 *
AgNPs-MB	1.56/12.79	0.78/0.79	0.78/0.79	0.78 */0.79 *
AgNPs-NMBN	1.56/13.91	0.39/0.43	0.39/0.21	0.39 */0.10 *
AgNPs-NMBN-Zn	1.56/16.66	0.39/0.26	0.39/0.26	0.39 */0.26 *

AgNO_3_, silver nitrate; AgNPs, silver nanoparticles; MB, methylene blue; NMBN, new methylene blue N; NMBN-Zn, new methylene blue N Zinc; *, concentration (µg mL^−1^) used in antifungal survival test.

**Table 3:** Fractional inhibitory concentration index (FICI) of APDT with AgNPs-PS in *C. albicans*.

Compound/ Radiant Exposure	5 J cm^−2^		10 J cm^−2^		15 J cm^−2^	
	FICI	Effect	FICI	Effect	FICI	Effect
AgNPs-MB	2.0	indifferent	1.5	indifferent	1.25	indifferent
AgNPs-NMBN	9.0	antagonistic	0.26	synergistic	0.5	synergistic
AgNPs-NMBN-Zn	0.3	synergistic	0.5	synergistic	0.3	synergistic

AgNPs, silver nanoparticles; MB, methylene blue; NMBN, new methylene blue N; NMBN-Zn, new methylene blue N Zinc.

**Table 4:** Fractional inhibitory concentration index (FICI) of AgNPs-PS in *F. keratoplasticum*.

Compound/ Radiant Exposure	5 J cm^−2^		10 J cm^−2^		15 J cm^−2^	
	FICI	Effect	FICI	Effect	FICI	Effect
AgNPs-MB	0.37	synergistic	0.6	additive	0.6	additive
AgNPs-NMBN	0.56	additive	0.30	synergistic	0.5	synergistic
AgNPs-NMBN-Zn	0.18	synergistic	0.31	synergistic	1.1	indifferent

AgNPs, silver nanoparticles; MB, methylene blue; NMBN, new methylene blue N; NMBN-Zn, new methylene blue N Zinc.

**Table 5:** Doses of compounds injected in *Galleria mellonella*.

Compound (mg kg^−1^)	Group of Larvae				
	1	2	3	4	5
AgNO_3_	0.31	0.62	1.25	2.5	5.0
AgNPs	0.31	0.62	1.25	2.5	5.0
MB	1.3	2.6	5.2	10.41	20.83
NMBN	1.64	3.28	6.57	13.15	26.31
NMBN-Zn	1.04	2.08	4.16	8.3	16.6
AgNPs-MB	0.31/1.13	0.62/2.6	1.25/5.20	2.5/10.4	5.0/20.83
AgNPs-NMBN	0.31/1.64	0.62/3.28	1.25/6.57	2.5/13.15	5.0/26.31
AgNPs-NMBN-Zn	0.31/1.04	0.62/2.08	1.25/4.16	2.5/8.3	5.0/16.6

AgNO_3_, silver nitrate; AgNPs, silver nanoparticles; MB, methylene blue; NMBN, new methylene blue N; NMBN-Zn, new methylene blue N Zinc.

## Data Availability

Data is contained within the article or supplementary material.

## Supplementary Materials

The following are available online at https://www.mdpi.com/article/10.3390/antibiotics10050569/s1, Figure S1: Toxicity of APDT in *G. mellonella* larvae.
