# Supplementary material for: Phenothiazinium Photosensitizers Associated with Silver Nanoparticles in Enhancement of Antimicrobial Photodynamic Therapy
Source: Antibiotics (Basel). 2021 May 12;10(5):569. doi: 10.3390/antibiotics10050569 (PMC8150796; doi:10.3390/antibiotics10050569)
Supplement: Supplementary file 1 [file antibiotics-10-00569-s001.zip › antibiotics-1185035-supplementary/antibiotics-1185035-supplementary.pdf]

Supplementary Material

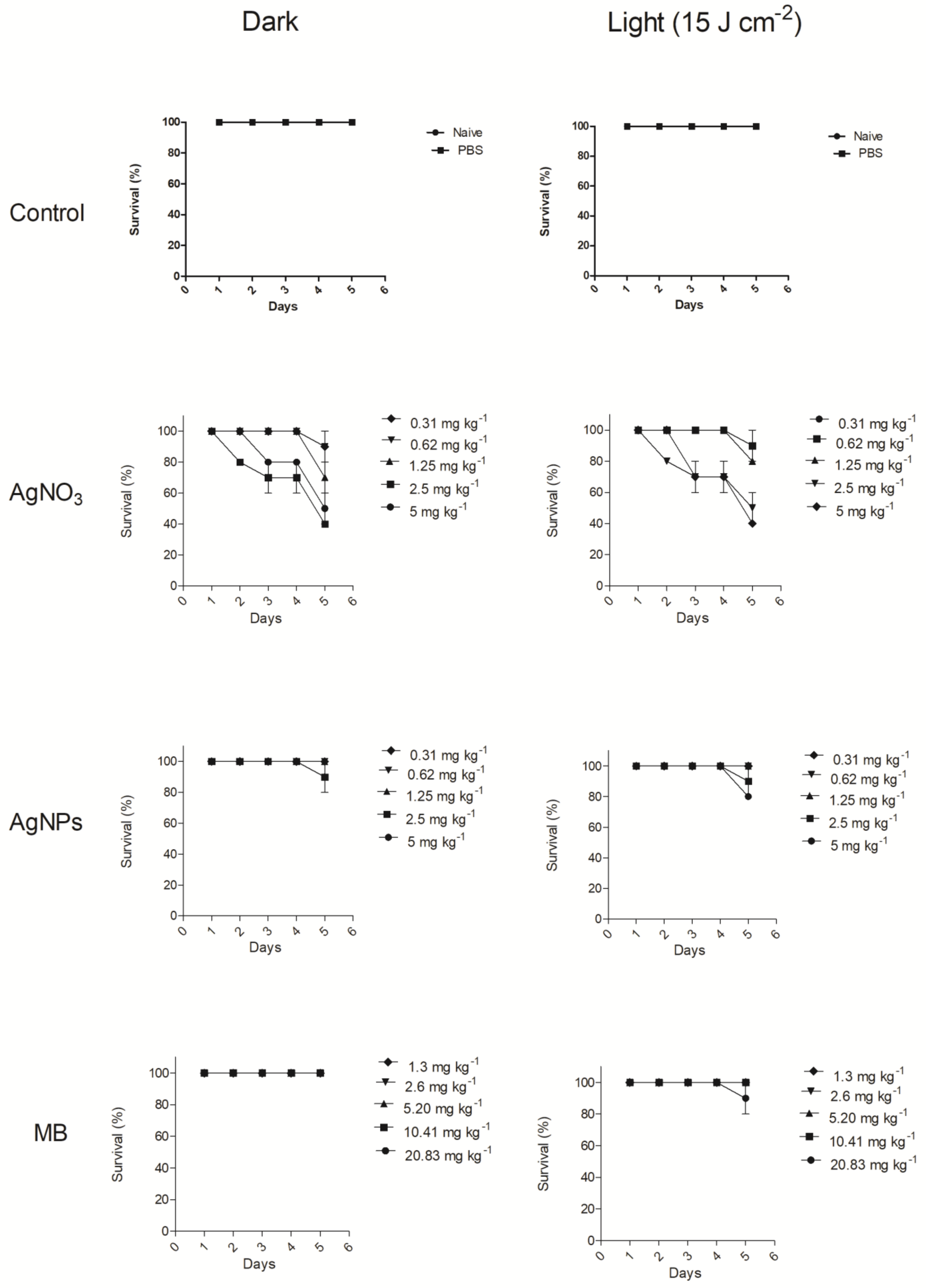

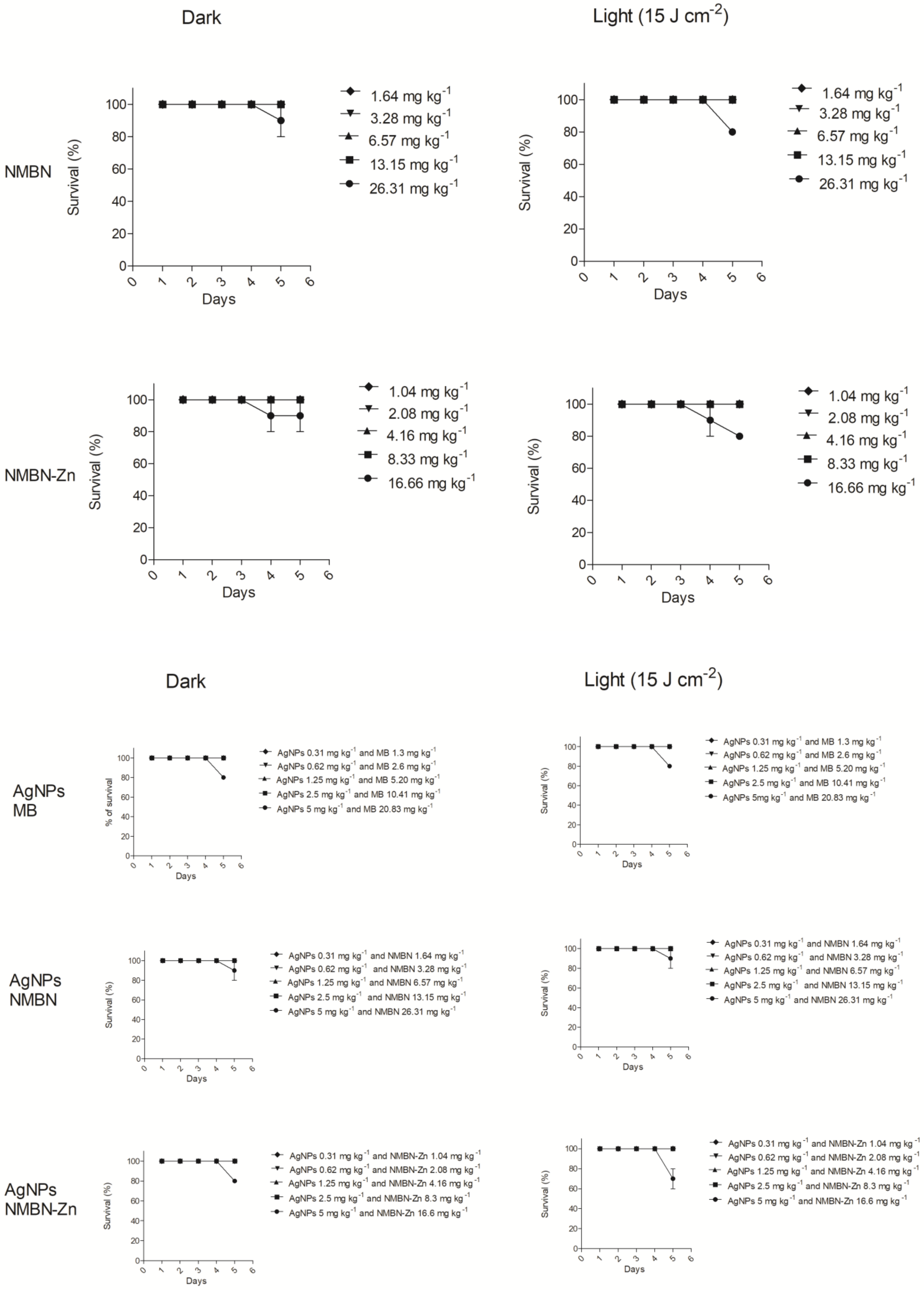
Dark
Light (15 J cm^{-2})
NMBN
Survival (%)
Days
1.64 mg kg^{-1}
3.28 mg kg^{-1}
6.57 mg kg^{-1}
13.15 mg kg^{-1}
26.31 mg kg^{-1}
NMBN-Zn
1.04 mg kg^{-1}
2.08 mg kg^{-1}
4.16 mg kg^{-1}
8.33 mg kg^{-1}
16.66 mg kg^{-1}
AgNPs MB
% of survival
AgNPs 0.31 mg kg^{-1} and MB 1.3 mg kg^{-1}
AgNPs 0.62 mg kg^{-1} and MB 2.6 mg kg^{-1}
AgNPs 1.25 mg kg^{-1} and MB 5.20 mg kg^{-1}
AgNPs 2.5 mg kg^{-1} and MB 10.41 mg kg^{-1}
AgNPs 5 mg kg^{-1} and MB 20.83 mg kg^{-1}
AgNPs 5mg kg^{-1} and MB 20.83 mg kg^{-1}
AgNPs NMBN
AgNPs 0.31 mg kg^{-1} and NMBN 1.64 mg kg^{-1}
AgNPs 0.62 mg kg^{-1} and NMBN 3.28 mg kg^{-1}
AgNPs 1.25 mg kg^{-1} and NMBN 6.57 mg kg^{-1}
AgNPs 2.5 mg kg^{-1} and NMBN 13.15 mg kg^{-1}
AgNPs 5 mg kg^{-1} and NMBN 26.31 mg kg^{-1}
AgNPs NMBN-Zn
AgNPs 0.31 mg kg^{-1} and NMBN-Zn 1.04 mg kg^{-1}
AgNPs 0.62 mg kg^{-1} and NMBN-Zn 2.08 mg kg^{-1}
AgNPs 1.25 mg kg^{-1} and NMBN-Zn 4.16 mg kg^{-1}
AgNPs 2.5 mg kg^{-1} and NMBN-Zn 8.3 mg kg^{-1}
AgNPs 5 mg kg^{-1} and NMBN-Zn 16.6 mg kg^{-1}

Figure S1. Toxicity of APDT in *G. mellonella* larvae. Larvae were inoculated with the compounds AgNO3, AgNPs, MB, NMBN, NMBN-Zn, and associations. Left, Survival of *G. mellonella* larvae over the days in the dark; Right, survival of *G. mellonella* larvae over the days after APDT 15 J cm^{-2}. Control, naïve and PBS; AgNO3, silver nitrate; AgNPs, silver nanoparticles; MB, methylene blue; NMBN, new methylene blue N; NMBN-Zn, new methylene blue N Zinc; associa-tions AgNPs-MB, AgNPs-NMBN, and AgNPs-NMBN-Zn.
